# Thymic stromal lymphopoietin promotes human eosinophil-basophil lineage commitment: a key role for tumor necrosis factor-alpha

**DOI:** 10.1186/1710-1492-10-S1-A51

**Published:** 2014-03-03

**Authors:** Claudia CK Hui, Sina Rusta-Sallehy, Delia Heroux, Judah A Denburg

**Affiliations:** 1 Department of Medicine, McMaster University, Hamilton, ON, Canada

## Background

Allergic diseases are characterized by tissue eosinophilic and basophilic inflammation. Both epithelial-derived thymic stromal lymphopoietin (TSLP) and eosinophil/basophil (Eo/B) lineage-committed progenitor cells are upregulated and found at sites of allergic inflammation [1-3]. We have previously shown that TSLP mediates the differentiation of peripheral blood (PB) CD34^+^ progenitor cells into eosinophils and basophils. However, the specific mechanisms through which TSLP promotes this lineage commitment are unclear. The aim of this study is to characterize the intracellular mechanisms by which TSLP mediates Eo/B differentiation.

## Methods

Purified PB CD34^+^ progenitors were stimulated overnight with media, IL-3 (1ng/mL), TSLP (10ng/mL), or IL-3/TSLP and assessed for cytokine and chemokine secretion using Luminex assays. Alterations in Eo/B colony forming units (CFU) and surface expression of TSLPR post-stimulation with IL-3/TSLP (and/or neutralizing anti-TNFα Ab) were assessed by methylcellulose cultures and flow cytometry respectively.

## Results

TSLP alone induced significant levels of IL-1β, IL-6, TNFα, and CXCL8 from PB CD34^+^ cells, compared to unstimulated controls (p<0.05). IL-3/TSLP-stimulated CD34^+^ cells released significant levels of IL-1β, IL-6, IL-13, TNFα, CXCL8 and CCL2, but failed to secrete detectable levels of IL-4, IL-9, GM-CSF, IFNγ, and eotaxin. Blockade of TNFα *in vitro* in the differentiation assays inhibited both TSLPR expression (p<0.05) and IL-3-responsive Eo/B CFU formation (p<0.05). Overnight stimulation of PB CD34^+^ cells with IL-3 (10ng/mL) and TNFα (50pg/mL) enhanced surface expression of TSLPR to comparable levels post TSLP/IL-3-stimulation. Moreover, pre-stimulating CD34^+^ cells with IL-3/TNFα prior to culturing in methylcellulose cultures resulted in enhanced sensitivity to TSLP-mediated Eo/B colony formation at lower concentrations of TSLP.

## Conclusions

We have previously shown that stimulation of human PB CD34+ cells with TSLP promotes Eo/B differentiation through upregulation of IL-3Rα and TSLPR. Our current study demonstrates that TSLP can modulate Eo/B lineage commitment, by inducing PB CD34^+^ cells to actively secrete chemokines and cytokines (key among which is TNFα), which, together with IL-3, induce the upregulation of TSLPR, leading to the subsequent amplification of Eo/B CFU. The novel role of TSLP-induced Eo/B differentiation points to the importance of the epithelium, and its responses to environmental stimuli, in the development of allergic diseases.
